# Increasing the Offer, Shifting the Offer: Patients' Perspectives on Routinely Offering HIV Counseling and POC Testing in the Health Services Program of an Urban Community Health Centre

**DOI:** 10.3389/fpubh.2020.00053

**Published:** 2020-03-18

**Authors:** Lynne Elizabeth Leonard, Sarah Vannice, Lindsay Wilson, Celia McCellan, Candis Lepage

**Affiliations:** HIV and HCV Prevention Research Team, School of Epidemiology and Public Health, Faculty of Medicine, University of Ottawa, Ottawa, ON, Canada

**Keywords:** HIV counseling and testing, reaching the undiagnosed, routine offer, provider-initiated, point of care, acceptability of the offer, venue diversification, Community Health Centre patients

## Abstract

**Objectives:** Canadian epidemiologic data demonstrate the fallibility of established HIV testing approaches to reach, diagnose, and link to care a significant portion of the population thereby contributing to missed opportunities to reduce onward HIV transmission. Increasing and diversifying entry points to accessing HIV testing may be a successful strategy to reach people who remain undiagnosed. We sought to determine the perspectives of patients on the acceptability of an offer of routine non-targeted provider-initiated HIV counseling and point-of-care (POC) testing in the health services program of a Community Health Centre in downtown Ottawa, the capital of Canada.

**Methods:** Patients aged 18 years and over accessing the Health Services Program for scheduled clinical appointments were approached by research staff with the offer of a POC HIV test with pre- and post-test counseling. All patients accepting the offer and those declining the offer were offered the opportunity to complete an Acceptability Questionnaire.

**Results:** Questionnaire responses from eligible patients over four consecutive weeks in 2018 strongly endorse the acceptability of an offer of an HIV test in the context of their scheduled health services appointment for a separate clinical condition. This contention held both for those patients accepting the offer and proceeding to testing and for those patients declining the offer.

**Conclusions:** The perspectives of the patients in our study demonstrate that a routine offer of non-targeted provider-initiated HIV counseling and POC testing was considered not only to be an acceptable, but also an appropriate and welcome intervention in a community health services program. These results suggest the potential for actively engaging more individuals—including those less likely to be engaged through a targeted testing approach—in the documented benefits of the HIV care and treatment cascade by increasing the HIV test offer through routine provider initiation. In addition, at the population level, shifting the offer through venue diversification, similarly shows potential for reducing engagement in ongoing HIV transmission behaviors and practices attributed to those unaware of their HIV positive status. Both outcomes fundamental to the goal of eliminating AIDS by 2030.

## Introduction

Ongoing HIV transmission remains a significant challenge to the health of people in Canada. The considerable proportion over a number of years, of people in Canada unknowingly living with HIV suggests a failure of established Canadian testing approaches to reach, diagnose, and link to care a significant portion of the population, represents missed opportunities to reduce onward HIV transmission, and impedes Canada's progress toward the 90-90-90 global targets established by UNAIDS and the World Health Organization (1). As the focal entry point to the HIV treatment cascade, to the sequence of activities that if followed can potentially lead to viral suppression of HIV in individuals (2, 3), implementing maximally accessible and acceptable HIV testing approaches is clearly indicated if Canada is to continue to work toward eliminating AIDS as a public health threat by 2030.

Early data from the Public Health Agency of Canada (PHAC) estimate that 16,000 people (range 13,000–19,000) were living with undiagnosed HIV in Canada at the end of 2014. That is, of the estimated 75,500 people living with HIV (including AIDS) in Canada in 2014, 21% were unaware of their infection. Retrospectively applying the methods employed in obtaining the 2014 estimate to 2011 data, PHAC reports a similar estimated proportion undiagnosed for 2011, approximately 21% (4). The latest data from PHAC indicate a lower estimate of 14% of the 63,100 people (plausible range 55,500–70,720) living with HIV (including AIDS) in Canada at the end of 2016; that is, one in every seven Canadians living with HIV had not been diagnosed, were unaware of their infection. PHAC cautions however, that as new infections are occurring at a rate greater than the number of deaths, the overall number of Canadians living with HIV is likely to increase in the years to come highlighting the need for innovative strategies to reach those people unaware of their HIV positive status (5).

These data are clearly of concern both absolutely and relatively. An array of serious and multi-level consequences is associated with the failure to reach this population. At the individual level there are significant treatment benefits associated with early access to HIV counseling and testing; antiretrovirals have demonstrated greater degrees of efficacy when started early in the course of infection before the immune system is too severely damaged. Conversely, delays in treatment access are associated with poorer patient outcomes, significant increases in opportunistic infections, decreased likelihood of immune recovery and diminished life expectancy; life expectancy is strongly related to CD4 count at the start of therapy (6–11).

In addition to these severe negative individual consequences, there are significant negative resource implications for public health and the health care system as a result of late presentation to care; health care costs are inversely related to CD4 cell counts. It has been estimated that the annual cost for a patient with a CD4 count of <200 cells/mm3 is approximately twice that of a patient whose counts are >500 cells/mm3 (12). A more recent study documented that significantly higher costs are incurred for those presenting to care with CD4 counts <350 cells/ml compared to individuals with higher CD4 counts and that these higher costs are sustained beyond the first year of care (13).

At the population level, lack of knowledge of personal positive HIV status has implications for ongoing transmission of HIV. A disproportionate number of HIV transmissions originate from people unaware of their infection (14, 15) as they are less likely to engage in HIV prevention measures (16) and, in the absence of ongoing treatment, are more likely to have a higher viral load (17, 18). Conversely, people aware of their HIV positive status are more likely to adopt strategies and to engage in practices to reduce the possibility of onward transmission (19–22).

Based on the premise that undiagnosed HIV infections in Canada represent a significant public health challenge, the Public Health Agency of Canada (PHAC) released a HIV Screening and Testing Guide in 2013 foregrounding a new programmatic approach to respond to this challenge. The previously existing HIV testing paradigm is best characterized as a targeted approach whereby HIV counseling and testing is offered to those Canadian populations considered to be disproportionally affected by HIV infection^1^. In contrast, the main tenet of this new evidence-based guide was to facilitate and support the normalization of HIV testing with the recommendation that “*the consideration and discussion of HIV testing be made a component of periodic routine care”* (23).

PHAC recommends this testing paradigm to reduce structural barriers inherent in a targeted risk-based or priority population approach to testing. Specifically, the Agency recommends a routine provider-initiated approach to reduce the prevalence of missed opportunities for testing occasioned by a health care provider's inability to accurately assess levels of risk for exposure to HIV and by some providers' lack of knowledge about HIV transmission and acquisition (23). HIV counseling and testing would be routinely offered without providers needing to assess the probability of a patient's or client's engagement in a specific HIV-related risk behavior or practice. With the provider routinely initiating the offer of HIV counseling and testing, the client or patient does not need to disclose personal behaviors or report stigmatizing and criminal practices, both of which mitigate against personally requesting an HIV test.

In this paper we examine the application of PHAC's recommendation of including the routine offer of HIV testing as part of a patient's routine clinical care. We report on the opinions of health services patients toward the concept of integrating routine non-targeted provider-initiated HIV counseling and POC testing in a health services program's standard of care.

## Materials and Methods

### Study Site

Sandy Hill Community Health Centre is a large, multi-component primary health care centre in downtown Ottawa which integrates primary care with mental health and substance use disorder services and treatment for patients across socio-economic status and severity of disorder.

### Study Procedures

All patients aged 18 years and over accessing the Health Services Program for scheduled clinical appointments were eligible to participate in the research with the exception of patients presenting with the following profiles consistent with eligibility criteria reported in the literature: patients for whom the offer would interfere with the assessment and treatment of their chief complaint (24, 25); patients for whom the offer would prolong their visit (24, 25); patients unable to provide informed consent including individuals with an altered mental status or who could not communicate sufficiently in a language necessary for being informed about the testing process (26–29); patients presenting with an urgent care need (26, 28, 29); and those who were deemed to be in too much pain to proceed with the procedures associated with the offer (27).

Patients were informed of the research study on registration by the Centre's administrative staff who distributed information cards describing the study and explained that research team members would routinely approach patients in the waiting room to discuss the project and to determine eligibility. Research staff explained that following their clinical appointment, the test and pre- and post-test counseling would take place in a private room where specifically trained research staff would discuss an informed consent document before proceeding with counseling and the performance of the test; results would be available before they left the Centre. Patients with a non-reactive test result would be shown the results of the test, be engaged in post-test counseling and offered promotional material. Patients with a preliminary positive or indeterminate test result (confirmed by a second member of the research or clinical team) would be able to speak with clinical personnel who would offer support, the opportunity to undergo a blood draw and, with their consent, to initiate referrals to the HIV specialist physician on site. Patients not yet ready to undergo the confirmatory blood test would be encouraged to make an appointment with an HIV specialist or to return to the Centre for further discussion at an appropriate time and were given details of resources and personnel available.

Patients who were approached and who declined the offer of undertaking the POC HIV test were offered the opportunity to complete an anonymous Acceptability Questionnaire. Questionnaires were placed by these patients in a sealed envelope and left in a box which was emptied on a daily basis. Patients accepting the offer of the test were also offered the opportunity, as appropriate, to complete the questionnaire and place it in the sealed envelope provided to be collected by the research staff member performing the test before the patient returned to the waiting room.

### Study Materials—HIV POC Testing Device

Point-of-care (POC) or rapid testing requires a few drops of blood from a finger prick thereby eliminating concerns around venipuncture. Additional benefits of this POC model for the patient are widely considered to be the rapid turnaround of results (negative and preliminary positive) and the opportunity to undertake pre- and post-test counseling at the same visit and with the same person. It therefore allows for continuity of the counseling experience which is much more difficult to ensure with the two-week waiting period that comes with standard HIV testing (30, 31). Patient satisfaction with POC testing has been documented to be high among both individuals who tested negative and those who tested positive for HIV and the vast majority of patients in several studies expressed confidence in its accuracy (24, 28).

The INSTI HIV-1/HIV-2 Rapid Antibody Test (bioLytical Laboratories, Canada) was licensed by Health Canada in 2005 for use by health care providers in undertaking POC testing (32). Sensitivity and specificity are comparable to current laboratory screening tests (99.6% sensitivity and 99.3% specificity) (23, 31). Introduced in Canada specifically to reduce barriers to early diagnosis and increase access to timely care, these testing materials were used in our study applying the policies, procedures and quality of assurance measures developed by the Ontario Ministry of Health and Long Term Care (33). The training for the members of the research team on the use of the INSTI HIV-1/HIV-2 Antibody Test was based on the Quality Assurance Program for POC testing (33), and was undertaken to ensure the delivery of consistently high quality, accurate and efficient HIV test results.

### Study Materials—Study Instrument

The introduction to the acceptability questionnaire detailed the reasons the research was being undertaken and by whom; the fact that the questionnaire was voluntary and anonymous; that people could choose not to answer any questions; that their decision to complete or decline to complete the questionnaire would not affect their care at the Centre; and that completion of the questionnaire would be taken as indicating their consent to participate in the research. The study received ethical approval from the Office of Research Ethics and Integrity at the University of Ottawa.

The questionnaire covered the study outcomes of interest: each patient's level of agreement or disagreement with statements concerning how they felt about being approached with the offer of HIV counseling and testing; the degree to which an array of factors affected their decision to accept or decline testing; their self-assessed risk of acquiring HIV; and their history of engagement with clinical physicians in the previous year. Those patients who went on to testing answered questions concerning their HIV testing history and results, and their level of agreement or disagreement with statements about their experience of the test undertaken through the research project. All patients were offered the opportunity to answer brief socio-demographic questions.

In this paper we report on the first two outcomes—the acceptability and appropriateness of an offer of HIV counseling and testing in the context of a clinical appointment arranged for a separate medical issue from the perspectives of patients both accepting and declining the offer. Understanding how patients perceive the routine offer of a rapid HIV test with pre-and post-test counseling may help to increase acceptance of testing for HIV and enable patient-relevant policy and program redevelopments. A separate paper reports on variables significantly associated with accepting the offer of an HIV test in the study and those patients' experiences with particular aspects of the POC test.

### Analysis

Using SPSS 25 software (IBM, SPSS Statistics) the two populations of patients, those accepting the offer of HIV testing and those declining, were compared with chi-square and Fischer exact tests as appropriate. Significance was set at *p* ≤ 0.05.

## Results

The study was carried out over 16 consecutive weekdays in January and February 2018. Over the 4 weeks of data collection an average of 150 patients per week attended the health services program for scheduled clinical appointments. Respecting the established eligibility parameters, research staff approached 44% of these patients in week 1, 82% in week 2, 41% in week 3, and 24% in week 4.

All 31 patients who accepted the offer of the test and went on to testing completed the acceptability questionnaire. In addition, 64 patients who declined the offer of the test completed the questionnaire. Data for this paper are extracted from these 95 completed questionnaires.

### Patient Profile

The mean age of the patients completing these questionnaires was 51 years and the majority self-identified as white heterosexual women. There were no significant differences on these sociodemographic variables between those patients who accepted the offer of the test and those who declined. Variability was however, observed in terms of patients' self-assessed risk of acquiring HIV. Although not achieving statistical significance, patients who did accept the offer on average rated their risk of HIV acquisition higher than those declining the test (Table 1).

**Table 1:** Profile of Patients Completing Acceptability Questionnaire.

**Characteristic**	**Total sample (*N* = 95)**	**HIV Point-of-care Testing offer**		***P*-value**
		**Accepted (*N* = 31) *n* (%)**	**Declined (*N* = 64) *n* (%)**	
Age, year				
Mean (Range)	51 (19–95)	51 (19–73)	51 (22–95)	0.84
	***n*** **=** **88**	***n*** **=** **31**	***n*** **=** **57**	
Gender				0.84
Male	30 (34.0)	11 (35.5)	19 (33.3)	
Female	58 (66.0)	20 (64.5)	38 (66.6)	
Transgender (MTF^a^)	0 (0.0)	0 (0.0)	0 (0.0)	
Transgender (FTM^a^)	0 (0.0)	0 (0.0)	0 (0.0)	
	***n*** **=** **85**	***n*** **=** **31**	***n*** **=** **54**	
Race^b^				
Arabic	3 (3.5)	1 (3.2)	2 (3.7)	1.00
Black	3 (3.5)	2 (6.5)	1 (1.9)	0.28
Indigenous	3 (3.5)	1 (3.2)	2 (3.7)	1.00
White	71 (83.5)	26 (83.9)	45 (83.3)	0.78
Other	6 (7.0)	2 (6.5)	4 (7.4)	1.00
	***n*** **=** **85**	***n*** **=** **30**	***n*** **=** **55**	
Sexual Orientation				0.58
Straight/heterosexual	75 (88.2)	28 (93.3)	47 (85.5)	
Gay/homosexual/lesbian	3 (3.5)	1 (3.3)	2 (3.6)	
Bisexual	4 (4.7)	0 (0.0)	4 (7.3)	
Pan-sexual/two-spirit	2 (2.4)	1 (3.3)	1 (1.8)	
Other	1 (1.2)	0 (0.0)	1 (1.8)	
	***n*** **=** **85**	***n*** **=** **28**	***n*** **=** **57**	
Self-assessed Personal Risk of Acquiring HIV Mean (1 = No risk, 5 = High risk)	1.55	1.79	1.44	0.09
	***n*** **=** **86**	***n*** **=** **29**	***n*** **=** **57**	
Attended Sandy Hill CHC in the past year	71 (82.6)	26 (89.7)	45 (78.9)	0.22
	***n*** **=** **58**	***n*** **=** **19**	***n*** **=** **39**	
Mean number of visits to Sandy Hill CHC in past year	9.05 (1–156)	7.6 (1–26)	9.7 (1–156)	0.72
	***n*** **=** **85**	***n*** **=** **28**	***n*** **=** **57**	
Reported having a family physician	81 (95.3)	27 (96.4)	54 (94.7)	1.00
	***n*** **=** **63**	***n*** **=** **18**	***n*** **=** **45**	
Mean number of visits to family physician in past year	3.3 (1–20)	4.3 (0–20)	2.9 (0–12)	0.16
	***n*** **=** **86**	***n*** **=** **29**	***n*** **=** **57**	
Plan to take a HIV test in the future	33 (38.4)	14 (48.3)	19 (33.3)	0.02

a *MTF, Male-to-Female; FTM, Female-to-Male*.

b *Proportions surpass 100% as 1 participant who accepted the HIV POC testing offer identified as belonging to more than one race*.

### Patients' Perspectives on Being Offered an HIV POC Test at Their Health Services Appointment

The vast majority of the patients who completed the acceptability questionnaire agreed or strongly agreed that: they were pleased to be offered HIV testing at their health centre visit (84%); they understood they had a choice to accept or decline the offer (95%); they were comfortable discussing the offer of the test (84%); and they felt respected by the person offering the HIV testing (89%). In terms of understanding the parameters of the testing process, the majority of patients agreed or strongly agreed that it was very clear how long the test would take (76%) and that they were told what would happen during the HIV testing process (63%).

The vast majority of patients disagreed or strongly disagreed with the statements: my health centre visit is not the right time to be offered HIV testing (80%); I was upset that I was offered HIV testing today (89%); I felt pressured to accept the offer of HIV testing (88%); and I was offended that I was offered HIV testing today (93%) (Table 2).

**Table 2:** Patients' Perspectives on Being Offered an HIV POC Test at their Health Services Appointment.

	**Strongly disagree**	**Disagree**	**Neither agree nor disagree**	**Agree**	**Strongly agree**
	***N* (%)**	***N* (%)**	***N* (%)**	***N* (%)**	***N* (%)**
I was pleased to be offered HIV testing at my health center visit today (*n* = 93)	3 (3.2)	0 (0)	12 (12.9)	28 (30.1)	50 (53.8)
My health center visit is not the right time to be offered HIV testing (*n* = 93)	39 (41.9)	35 (37.6)	12 (12.9)	5 (5.4)	2 (2.2)
I was upset that I was offered HIV testing today (*n* = 93)	59 (63.4)	24 (25.8)	4 (4.3)	3 (3.2)	3 (3.2)
I understood that I could accept the offer or decline the offer of HIV testing (*n* = 93)	3 (3.2)	0 (0)	2 (2.2)	21 (22.6)	67 (72.0)
I felt pressured to accept the offer of HIV testing (*n* = 91)	62 (68.1)	18 (19.8)	5 (5.5)	1 (1.1)	5 (5.5)
I was comfortable discussing the offer of the test (*n* = 93)	3 (3.2)	4 (4.3)	8 (8.6)	34 (36.6)	44 (47.3)
I was offended that I was offered HIV testing today (*n* = 91)	64 (70.3)	21 (23.1)	3 (3.3)	0 (0)	3 (3.3)
I felt respected by the person offering me HIV testing today (*n* = 93)	6 (6.4)	2 (2.2)	2 (2.2)	29 (31.2)	54 (58.0)
It was very clear how long the testing would take (*n* = 92)	2 (2.2)	5 (5.4)	15 (16.3)	33 (35.9)	37 (40.2)
I was told what would happen during the HIV testing (*n* = 92)	5 (5.4)	10 (10.9)	19 (20.6)	27 (29.3)	31 (33.8)

Disaggregating these data by acceptance of the offer as shown in Table 3 revealed that, although not significant, the proportion of patients expressing levels of agreement with the positive attributes of the offer were higher among those who accepted the offer and went forward with testing compared to those who declined the offer. Similarly, the proportion of patients expressing levels of disagreement with the negative attributes of the offer were higher among those who accepted the offer of the test compared to those who declined the offer, but again the differences were not significant.

**Table 3:** Patients' Perspectives on Being Offered an HIV POC Test at their Health Services Appointment by Decision to Test.

	**Accepted HIV POC testing**			**Declined HIV POC testing**			***P-*value**
	**Strongly disagree/disagree**	**Neutral**	**Strongly agree/agree**	**Strongly disagree/disagree**	**Neutral**	**Strongly agree/agree**	
	***N* (%)**	***N* (%)**	***N* (%)**	***N* (%)**	***N* (%)**	***N* (%)**	
I was pleased to be offered HIV testing at my health center visit today	0 (0)	2 (6.5)	29 (93.5)	3 (4.8)	10 (16.1)	49 (79.1)	*0.2*
I understood that I could accept the offer or decline the offer of HIV testing	0 (0)	0 (0)	30 (100)	3 (4.8)	2 (3.2)	58 (92)	*0.46*
I was comfortable discussing the offer of the test	1 (3.2)	3 (9.7)	27 (97.1)	6 (9.7)	5 (8.1)	51 (82.2)	*0.67*
I felt respected by the person offering me HIV testing today	1 (3.2)	1 (3.2)	29 (93.6)	7 (11.3)	1 (1.6)	54 (87.1)	*0.34*
My health center visit is not the right time to be offered HIV testing	26 (83.9)	4 (12.9)	1 (3.2)	48 (77.4)	8 (12.9)	6 (9.7)	*0.66*
I was upset that I was offered HIV testing today	28 (90.3)	2 (6.5)	1 (3.2)	55 (88.7)	2 (3.2)	5 (8.1)	*0.56*
I felt pressured to accept the offer of HIV testing	28 (90.3)	1 (3.2)	2 (6.5)	52 (86.7)	4 (6.7)	4 (6.6)	*0.88*
I was offended that I was offered HIV testing today	29 (93.6)	1 (3.2)	1 (3.2)	56 (93.3)	2 (3.3)	2 (3.3)	*1.00*
It was very clear how long the testing would take	0 (0)	1 (3.2)	30 (96.8)	7 (11.5)	14 (22.9)	40 (65.6)	*0.003*
I was told what would happen during the HIV testing	0 (0)	1 (3.2)	30 (96.8)	15 (24.6)	18 (29.5)	28 (45.9)	*<0.0001*

Significant differences were observed however among patients' perspectives on their understanding of the parameters of the testing process. A significantly higher proportion of patients who accepted the offer of the test compared with those who declined the offer agreed or strongly agreed that it was very clear how long the testing would take (97 vs. 66% *p* = 0.003) and that they were told what would happen during the HIV testing process (97 vs. 46% *p* ≤ 0.0001).

## Limitations

This study has embedded limitations which need to be acknowledged before discussing the data. The possibility of selection bias cannot be excluded, and the generalizability of these findings is therefore limited. For example, recruitment was restricted to patients who could understand sufficient English to complete the questionnaire and consent procedures, and testing could only take place after the patient's scheduled appointment to protect the physician's time. As appointments often ran late, patients originally agreeing to undertake the test were subsequently not able to complete testing due to time constraints. The availability of interpreters and provision of the questionnaire in languages other than English, and the ability to conduct the test on agreement to test may well have increased both the number of patients completing the questionnaire and the number that accepted the offer of the test.

However, in a domain of limited Canadian study, these data do represent a unique perspective and contribution to the study of the application of PHAC's recommended alternative approaches to HIV counseling and testing. A recent scoping review of HIV POC testing in Canadian settings based on 10 peer-reviewed articles and 17 gray literature reports confirms the positive and favored aspects of the POC test itself compared to conventional HIV testing approaches among a variety of populations. Our study's uniqueness lies in the fact that we concentrated on the acceptability of *being approached with the offer* of an HIV test rather than on the merits of the testing material. In addition, the authors of the review assigned a low quality assessment to the articles included in the review as most were observational studies which would not apply to our study (34).

## Discussion

PHAC's HIV Screening and Testing Guide was released five years prior to the implementation of our research project, yet it can be argued that that there is a paucity of evaluative evidence parameters of successful diversification of HIV testing approaches and strategies, and on their acceptability in a variety of venues. This is a key evidence gap in working toward reducing the number of Canadians living with undiagnosed HIV infection with the associated negative clinical impacts both at the individual and at the population level.

If PHAC's recommended strategy is to be universally adopted, what essential programming and policy components can be extracted from our research to facilitate this process?

In terms of diversification of HIV testing approaches, a provider-initiated approach was overwhelmingly endorsed by our participants, the vast majority of whom reported they were pleased to be offered HIV testing, were not upset by being offered the opportunity to test, were comfortable discussing the offer of the test, and understood they could accept or decline the offer. A health care provider routinely offering HIV counseling and POC testing as a component of regular medical care removes the documented significant barriers experienced by an individual in requesting testing such as an amalgam of lack of comfort in discussing HIV testing; a reluctance and fear of reporting stigmatizing behavior and practices; and not having the pre-requisite knowledge to internalize personal HIV risk in order to be able to request HIV testing. Of importance and relevance to our current research, is the outcome of a research study examining the acceptability of an established tailored program of provider-initiated HIV counseling and testing—Ontario's prenatal HIV counseling and testing program. Many women—including those who received a positive HIV test result through the program—stated that they had not previously considered themselves to be at risk of HIV acquisition and thus would not have entertained testing for HIV had it not been offered (35, 36).

This documented importance and utility of routinely offering testing to those not necessarily seeking personal HIV testing is further reflected in our study results. The patients participating in the study by completing the questionnaire constituted a fairly homogeneous population. The vast majority were older white straight women; a population outside those considered as priority populations for whom HIV testing is recommended when a risk-based framework is applied. This is an interesting and unique observation and of importance when considering the demographics documented in the latest report of new HIV diagnoses in Canada. Of the total 2,402 new HIV diagnoses reported in Canada in 2017, 25% occurred among women, with heterosexual contact documented as the exposure category among the greatest proportion (61%) (37). In relation to the importance of the reported age of the patients offering their positive perspectives on the routine provider-initiated offer of HIV testing, older adults have been documented to be diagnosed later in the course of their HIV infection (38) and are a significant demographic in reports of new HIV diagnoses in Canada; among all reported HIV cases for 2017, the 50 years and older age group represented the second highest proportion at 23%. From the perspectives of the patients in our study, offering routine provider-initiated rather than risk-based HIV counseling and testing in an accessible, low threshold and frequently visited venue may represent a significant opportunity to engage this population in HIV testing and prevention behaviors and practices.

In terms of diversification of venues, from the perspectives of the patients in our study, it is clear that a Community Health Centre's Health Services Program is ideally positioned to offer HIV counseling and POC testing and offers an alternative to patients who might not attend more traditional HIV testing settings such as sexual health clinics. However, these patients were only informed of the study at the time of their attendance for a medical appointment at the Centre; these patients were not expecting to be in a position of deciding whether or not to undergo testing for HIV when attending for a separate medical issue. Acceptance of the offer of the test may well have been higher if the promotion of the benefits of the opportunity to undergo an HIV test and, through the use of the rapid POC test, to receive the results at the time of their health services appointment may well have increased the number of patients going on to testing. For example, Leber and colleagues (39) carried out a cluster-randomized control trial of the promotion of rapid testing for HIV in primary care among 40 general practices in London, England. The 20 practices in the intervention arm, which involved an educational outreach program promoting routine rapid HIV testing to new patients in general practice, documented higher rates of diagnosis of HIV compared to those practices undertaking an HIV test on patient request or offering a test based on a health care provider‘s assessment of risk. The rates of acceptance of the test in practices in the intervention arm of this study far exceeded our documented rates of acceptance which points to the potential utility of sustained promotion of the intervention.

Aligned with the increase in acceptance rates that could potentially be associated with promotion of the opportunity of HIV testing, our study results demonstrate the importance of a clear and detailed description of the testing process as a necessary facilitator of acceptance rates. The issues that mitigated against the patients in our study accepting the offer were lack of clarity as to how long the testing would take and what the test involved. These two statements were the only two that significantly differentiated those patients who accepted testing and those who declined testing and as such demonstrate compelling evidence of what is required from the perspectives of the patients to scale up provider-initiated POC testing for HIV.

## Conclusion

The Public Health Agency of Canada attributes the modeled phenomenon of a significant proportion of people in Canada living with HIV unaware of their diagnosis to “*a lack of testing and/or diagnosis.”* (40). Critically examining the application of diversification of availability of these opportunities and acceptability of alternate approaches to offering and carrying out an HIV test is clearly urgently indicated. The results from our unique study lend credence to the pursuit of further work in examining the implementation of tailored HIV counseling and POC testing programs in a medicalized community venue managed by dedicated and sole-tasked providers who do not need to assess the probability of engagement, or patients needing to acknowledge, a specific HIV-related risk behavior or practice. Current Canadian epidemiologic data point to the emergent and urgent necessity of such action.

## Data Availability Statement

The datasets for this article are not publicly available at this time as preparation of an additional article using these data is currently in progress. Requests to access the datasets should be directed to Lynne Leonard, lleonard@uottawa.ca.

## Ethics Statement

The study was reviewed and approved by the Office of Research Ethics and Integrity, University of Ottawa. Written informed consent for participation was not required for this study in accordance with the national legislation and the institutional requirements.

## Author Contributions

LL developed the original research study (design, methodology, questionnaire) with contributions to the methodology by SV, LW, and CM. CL completed the quantitative analysis. LL conceived the original idea for this manuscript and prepared the first draft. All authors had full access to the data, reviewed and revised the draft, approved the final version of the manuscript and agreed to publication.

### Conflict of Interest

The authors declare that the research was conducted in the absence of any commercial or financial relationships that could be construed as a potential conflict of interest.

## Footnotes

^1^Groups in Canada considered by the Public Health Agency of Canada to be disproportionately affected by HIV infection include gay, bisexual, and other men who have sex with men; people who share drug-using equipment; people from countries where HIV is endemic; and indigenous peoples.
